# Temporal patterns of gelatinous zooplankton distribution and environmental drivers in the south-western Mediterranean Sea

**DOI:** 10.3897/BDJ.11.e101790

**Published:** 2023-05-16

**Authors:** Ghollame Ellah Yacine Khames, Aldjia Kherchouche, Zakia Alioua, Aziz Hafferssas

**Affiliations:** 1 University of Sciences and Technology Houari Boumediene, Faculty of Biological Sciences- Laboratory of Biological Oceanography and the Marine Environment-Pelagic Ecosystem Team. BP 32, El Alia, Bab-Ezzouar, Algiers, Algeria University of Sciences and Technology Houari Boumediene, Faculty of Biological Sciences- Laboratory of Biological Oceanography and the Marine Environment-Pelagic Ecosystem Team. BP 32, El Alia, Bab-Ezzouar Algiers Algeria; 2 University of Sciences and Technology Houari Boumediene, Faculty of Biological Sciences- Laboratory of Biological Oceanography and the Marine Environment-Fisheries Team. BP 32, El Alia, Bab-Ezzouar, Algiers, Algeria University of Sciences and Technology Houari Boumediene, Faculty of Biological Sciences- Laboratory of Biological Oceanography and the Marine Environment-Fisheries Team. BP 32, El Alia, Bab-Ezzouar Algiers Algeria

**Keywords:** coastal Algeria, pelagic ecology, gelatinous species, environmental parameters, distribution and dispersal, Mediterranean Sea

## Abstract

This study aims to investigate the distribution of gelatinous zooplankton in relation to environmental parameters along the coastal regions of Algeria in the south-western Mediterranean Sea. A total of 48 species were recorded from nine sampling stations located in the central (Sidi Fredj) and western (Habibas Islands) sectors of the Algerian coast. The results showed that the seasonal distribution of gelatinous species exhibits significant variations. Amongst cnidarians, *P.noctiluca*, *M.atlantica* and *A.tetragona* are the most abundant species. Chaetognaths are primarily represented by *F.enflata* and *P.friderici*. Tunicates display high diversity, with *T.democratica*, *O.longicauda* and *D.nationalis* as the most abundant species. Lastly, in molluscs, *H.inflatus* and *L.trochiformis* are the most abundant species. The nMDS and ANOSIM analysis reveal significant differences in the ecological community structures between the Habibas Islands and Sidi Fredj. Redundancy analysis results show relationships between different marine species and environmental variables, such as temperature, chlorophyll a and salinity. The studied species exhibit positive or negative correlations with these variables, suggesting an influence of these factors on their abundance and distribution. This study enhances our understanding of the factors that govern the distribution and dispersal of gelatinous zooplankton in the Mediterranean Sea and has significant implications for predicting changes in the distribution of these species under future environmental scenarios.

## Introduction

Gelatinous zooplankton, including Ctenophora, hydromedusae, scyphomedusae, Siphonophora, Chaetognatha Appendicularia, Doliolida and Salpida, are amongst the most abundant planktonic organisms in marine food webs (Luo et al. 2020). The appearance of blooms of these organisms has been related to the fluctuations of environmental variables, such as advection displacement, temperature, salinity, light or nutrient supply (Chi et al. 2021). Such blooms have been reported in various marine areas, including the Black Sea (Grishin et al. 2007), the Red Sea (Sola et al. 2019), the Bering Sea (Brodeur et al. 1999), the coast of Japan (Purcell et al. 2007) and the Mediterranean Sea (Boero 2013). These blooms of gelatinous zooplankton play important roles in marine food webs by acting as both prey and predator (Wright 2019). Salps, a type of gelatinous zooplankton, are efficient non-selective gelatinous filter feeders and they are considered predators of different trophic levels (Licandro et al. 2006). Copepods have been reported to be the main food of Chaetognatha (Sanvicente-Anorve et al. 2020). Siphonophora occupy multiple trophic niches and prey on a diversity of taxa (Hetherington et al. 2022). Jellyfish, another type of gelatinous zooplankton, are important predators of fish eggs and larvae and their predation is believed to be the main factor determining fish recruitment. For example, in the case study of *Pelagicanotiluca* medusea and the anchovy *Engraulisencrasicolus*, jellyfish predation played a role in determining fish recruitment (Tilves et al. 2016).

Understanding how gelatinous zooplankton communities are influenced by environmental factors, such as local hydrography and physical forcing, can have important implications for fisheries and higher trophic predators (Haberlin et al. 2019). For the entire pelagic ecosystem, long-term fluctuations in gelatinous zooplankton communities can have far-reaching consequences (Licandro et al. 2006). However, anthropogenic global warming has resulted in a sustained increase in average temperature, causing a loss of biodiversity that will inevitably alter ecosystem functioning (Burrows et al. 2014). Marine phytoplankton and zooplankton form the backbone of the ocean's food web and are particularly vulnerable to ocean warming, forcing them to shift their distribution ranges polewards in search of optimal thermal habitats. However, the impacts of climate change on biodiversity are poorly understood (Benedetti et al. 2021).

Research on gelatinous zooplankton in Algerian waters is limited and incomplete, with a primary focus on copepods (Seridji and Hafferssas 2000, Hafferssas and Seridji 2010, Chaouadi and Hafferssas 2018). Few studies have investigated the gelatinous zooplankton communities in Algeria, in contrast to other regions like the Tunisian coast (Daly et al. 2003, Touzri et al. 2012), the Black Sea (Grishin et al. 2007) and the Moroccan coast (Aouititen et al. 2019), where studies have explored their distribution, abundance and impact on fish stocks. While there are some data available on the species composition and distribution of Medusae (Kherchouche and Hafferssas 2020), the other groups (such as Ctenophora, hydromedusae and scyphomedusae, Siphonophora, Chaetognatha Appendicularia, Doliolida and Salpida) have only been studied by Khames and Hafferssas (2019). Therefore, characterising the gelatinous zooplankton communities in Algeria is necessary to gain a better understanding of their ecological and temporal dynamics and the taxa they comprise. Given the documented impacts of anthropogenic climate change on marine ecosystems, it is likely that gelatinous zooplankton communities in Algeria will experience significant shifts in their distribution and abundance, with potential consequences for higher trophic levels and fisheries.

This study aims to address these gaps in knowledge by characterising the gelatinous zooplankton communities in two regions of the central and western sectors of Algeria, including the Habibas Islands, an important marine protected area and Sidi Fredj, a highly valued coastal zone. Two regions of the central and western sectors of Algeria were chosen for this investigation. In the west, the Habibas Islands are an important biological hotspot of marine and terrestrial biodiversity, classified as Specially Protected Areas under the Barcelona Convention framework (UNEP/MAP, SPA/RAC, SPAMIs 2020) and considered as a model example in terms of ecological status. However, Sidi Fredj covered a wide range of anthropogenic pressures in the central sector of the Algerian coast, including strong demographic expansions and coastal development of tourism and recreational ports, as well as recreational fishing and diving clubs. The study has the following main objectives: (1) to address the gap in knowledge regarding the gelatinous zooplankton communities along the Algerian coast, (2) to analyse their distribution in the Habibas Islands, which are an important Algerian marine protected area, (3) to compare their distribution with that of Sidi Fredj, a highly valued coastal zone in Algeria (south-western Mediterranean Sea) and (4) to gain a better understanding of how periodic changes in abiotic environmental conditions, such as temperature, salinity and chlorophyll a levels, influence the population dynamics of these communities.

## Material and methods

### Study area

**Figure 1.**
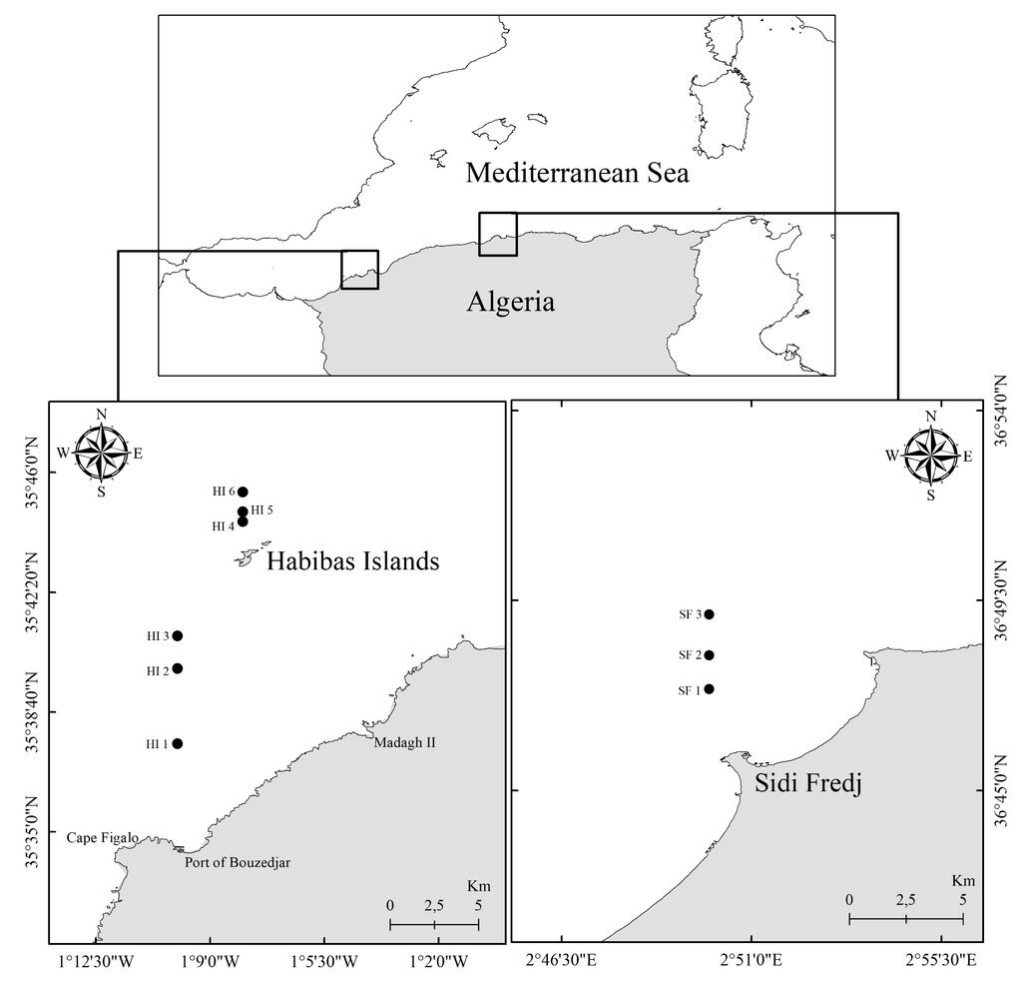
Study area with sampling stations of gelatinous zooplankton on the Algerian coast.

In the western sector of the Algerian coast, the Habibas Islands (HI) are located 26 miles (41.8 km) from Cape Figalo west of Oran, 10 miles (16.1 km) from the port of Bouzedjar and 5.8 miles (9.3 km) from the nearest continental point at Madagh II. Sidi Fredj (SF) is located at the central sector of Algeria. It is exposed to strong demographic expansions and the coastal development of tourism activity and recreational ports (Fig. 1).

### Sample collection

The study involved collecting a total of 24 biological samples from two locations: Sidi Fredj and Habibas Islands. At Sidi Fredj, three samples were taken from each of the three stations during autumn, winter, spring and summer, resulting in a total of 12 samples. At Habibas, six samples were collected from each of the six stations during spring and summer, resulting in another 12 samples (Table 1). The biological samples were collected using a Working Party II (WP2) plankton net with a 200 μm mesh and a net mouth diameter of 57 cm. The net was lowered vertically from a depth of 60 metres to the surface and the samples were collected during each season from the designated stations at Sidi Fredj and Habibas Islands. To minimise damage to the animals, the samples were stored in a 7 litre bucket and immediately fixed with a formaldehyde solution (4%) (Kirschner 1985).

Under a Zeiss Stemi SV 6 (Germany) microscope, specimens were carefully examined and identified, based on appropriate taxonomic literature, including works by Tregouboff and Rose (1957), Fenaux (1967), Rampal (1975), Fenaux (1998), Boltovskoy (1999), Bouillon et al. (2006). The zooplankton counts were utilised to calculate the mean abundances (Abd) expressed in ind.m^−3^, as well as the standard deviation (SD).

Environmental parameters, including temperature, salinity and chlorophyll a, were mesured at a depth of 0 to 50 metres during zooplankton collections using a Niskin bottle. Temperature and salinity were measured at each sample using a multiparameter instrument (HI 9828-12202/Romania) at four depths (5, 15, 30 and 50 metres). However, we conducted the chlorophyll a assay in the laboratory using the Lorenzen technique (Aminot and Chaussepied 1983, Wright and Jeffrey 2006).

### Statistical analysis

The statistical analysis of the collected data was performed using R version 4.1.3 (R CORE TEAM 2022). In this study, analysis of variance (ANOVA) tests were employed to determine if there were any significant differences in the mean abundances of various groups across different seasons at Sidi Fredj and Habibas Islands. ANOVA is a statistical method that allows us to compare multiple groups while accounting for the variability within these groups.

The first step was to apply the non-metric Multidimensional Scaling ordination (nMDS) to show the distribution of gelatinous zooplankton samples on the Algerian coast in both regions. This was followed by a non-parametric analysis of similarities (ANOSIM) on species abundance to test whether there was a significant difference between the studied regions (Sidi Fredj and Habibas Islands) and the sampling periods (months, seasons) (Clarke et al. 2014).

To determine how the zooplankton community has changed over time relative to environmental variables, multivariate methods were used (Borcard et al. 2018). An initial detrended correspondence analysis (DCA) was applied to check if linear constrained ordination methods, such as redundancy analysis (RDA), were appropriate (Ter Braak and Šmilauer 2002). The length gradient was less than 3 units of standard deviation, indicating a homogeneous dataset for which linear constrained methods are appropriate.

## Results

### Environmental parameters

In May, the sea surface temperatures around the Habibas Islands were observed to fluctuate between 15.2°C and 19.9°C, while in July, they ranged from 17.1°C to 24.5°C. As for Sidi Fredj, the sea surface temperatures were relatively low during March (ranging from 15.3°C to 16°C) and April (ranging from 15.5°C to 16.4°C) and comparatively high in July (ranging from 17.9°C to 22.8°C) and November (ranging from 15.2°C to 18.5°C).

Regarding the sea surface salinity, values in the Habibas Islands ranged between 34 psu and 34.9 psu in May, while in July, they varied from 34.7 psu to 35.2 psu. In Sidi Fredj, the surface salinity was measured to be between 35.9 psu and 36.4 psu in March and between 35.8 psu and 36.1 psu in April, with a range of 35.7 psu to 36.5 psu in November. The maximum salinity was recorded in July, ranging from 36 psu to 36.7 psu.

**Figure 2.**
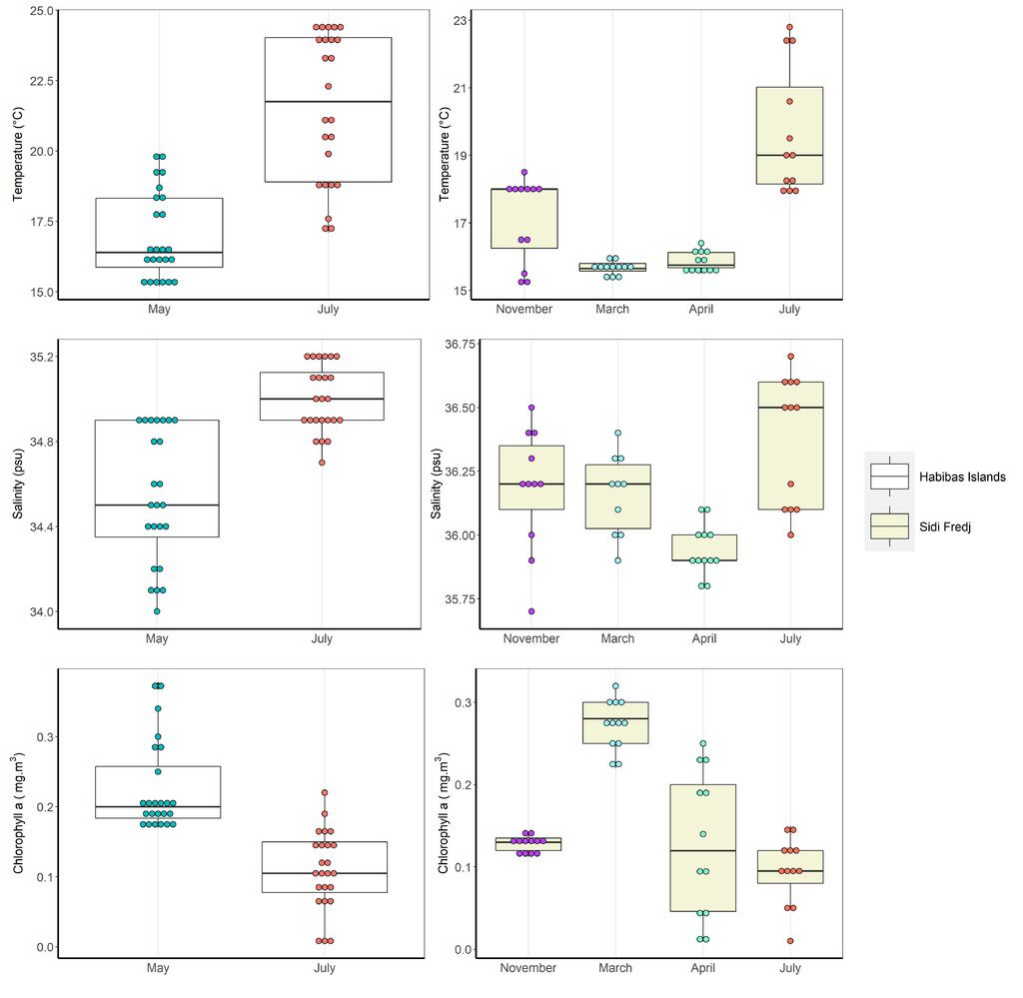
Boxplot of temperature, salinity and chlorophyll a of the surface layer (0-50 m) in Habibas Islands and Sidi Fredj.

Chlorophyll a recorded a maximum value at the Habibas Islands in May (ranging between 0.17 mg.m^-3^ and 0.37 mg.m^-3^). However, values were low in July (ranging between 0.008 mg.m^-3^ and 0.22 mg.m^-3^). At Sidi Fredj, chlorophyll a was highest in March (ranging between 0.22 mg.m^-3^ and 0.32 mg.m^-3^) and April (ranging between 0.01 mg.m^-3^ and 0.25 mg.m^-3^), followed by November (ranging between 0.11 mg.m^-3^ and 0.14 mg.m^-3^). The minimum chlorophyll a was registered in July with lower values (fluctuating between 0.01 mg.m^-3^ and 0.15 mg.m^-3^) (Fig. 2).

### Taxonomic diversity

**Figure 3.**
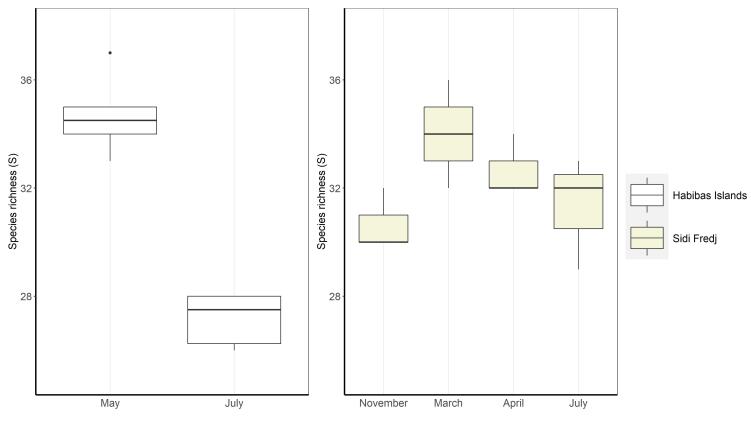
Boxplot of the species richness index of gelatinous zooplankton in Habibas Islands and Sidi Fredj.

The Habibas Islands and Sidi Fredj were found to harbour a total of 48 species of gelatinous zooplankton, belonging to five Chetognatha, six Mollusca, 13 Tunicata and 24 Cnidaria taxa (Table 2). Seasonal fluctuations in species richness were observed at both locations (Fig. 3). At the Habibas Islands, species richness appeared to be higher in May than in July, with a range of 33 to 37 species in May and 26 to 28 species in July. Meanwhile, the data collected at Sidi Fredj showed that species richness remains relatively stable during November, with a minimum and maximum of 30 and 32 species, respectively. In March and April, the species richness increased, with minimum and maximum values of 32 and 36 in March and 32 and 34 in April. However, in July, species richness was lower, with minimum and maximum values of 29 and 33, respectively.

### Seasonal variations in gelatinous zooplankton abundance

**Figure 4.**
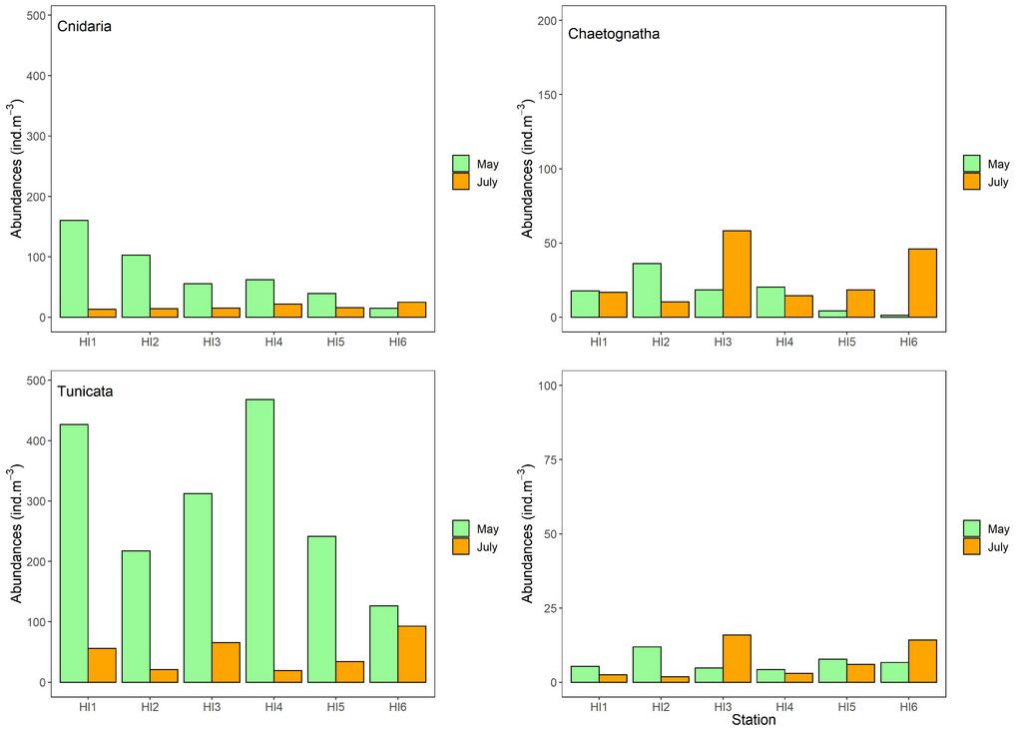
Distribution and variation of gelatinous zooplankton near Habibas Island.

In the Habibas Islands, different abundances of marine organisms were observed depending on the taxonomic groups and seasons. For Tunicata, a minimum abundance of 126 ind.m^-3^ (HI6) and a maximum abundance of 468 ind.m^-3^ (HI4) were exhibited in May. In July, the minimum abundance was 19 ind.m^-3^ (HI4) and the maximum abundance was 93 ind.m^-3^ (HI6). The ANOVA showed a statistically significant difference between seasons for Tunicata (P < 0.05). In May, Chaetognatha showed a minimum abundance of 1 ind.m^-3^ (HI6) and a maximum abundance of 36 ind.m^-3^ (HI2). In July, the minimum abundance of Chaetognatha was 10 ind.m^-3^ (HI2) and the maximum abundance was 58 ind.m^-3^ (HI3). The ANOVA showed that there was no statistically significant difference in the abundance of Chaetognatha between seasons (P > 0.05). Regarding Cnidaria, in May, their minimum abundance was 15 ind.m^-3^ (HI6) and the maximum abundance was 160 ind.m^-3^ (HI1). In July, a minimum abundance of 13 ind.m^-3^ (HI1) and a maximum abundance of 25 ind.m^-3^ (HI6) were noted. The ANOVA showed a statistically significant difference between seasons for Cnidaria (P < 0.05). For Mollusca, in May, the minimum abundance was 4 ind.m^-3^ (HI4) and the maximum abundance was 12 ind.m^-3^ (HI2). In July, the minimum abundance was 2 ind.m^-3^ (HI2) and the maximum abundance was 16 ind.m^-3^ (HI3). The ANOVA showed that there was no statistically significant difference in the abundance of Mollusca between seasons (P > 0.05) (Fig. 4).

**Figure 5.**
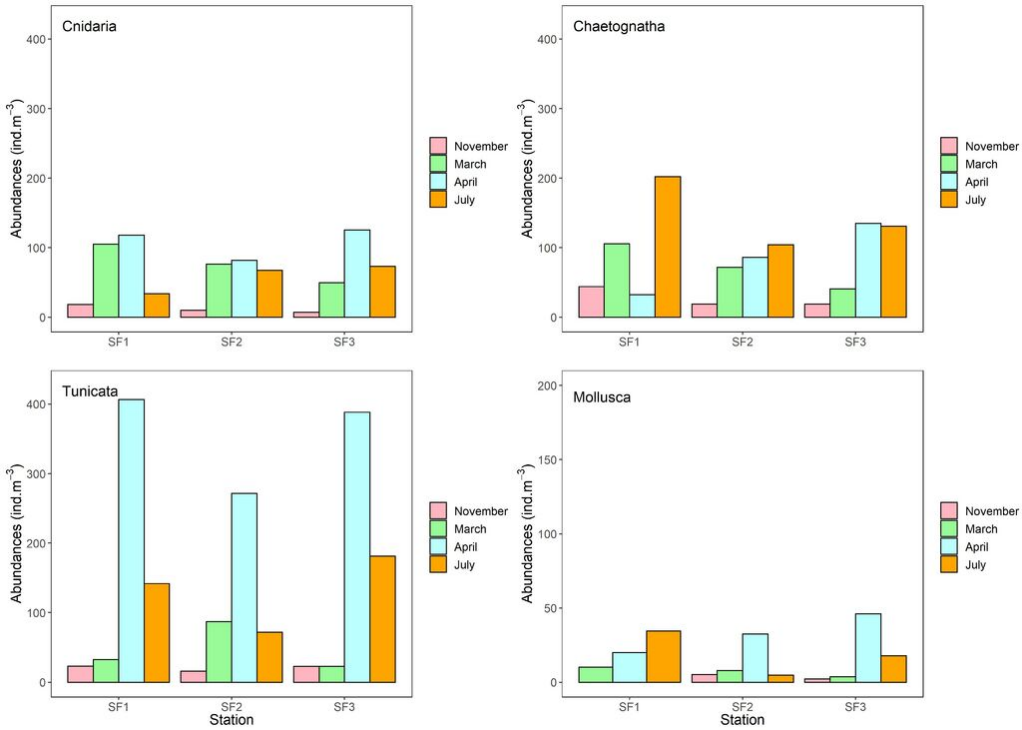
Distribution and variation of gelatinous zooplankton in Sidi Fredj.

At Sidi Fredj, a comprehensive analysis of the abundances of various groups across seasons demonstrated significant variations. ANOVA tests revealed significant differences (P < 0.05) in abundances between seasons within each group. For Chaetognatha, the highest abundance occurred in July at SF1 (202 ind.m^-3^), while the lowest was in November at SF2 (19 ind.m^-3^). In the case of Cnidaria, the peak abundance was observed in April at SF1 (118 ind.m^-3^), while the lowest occurred in November at SF3 (7 ind.m^-3^). For Mollusca, the maximum abundance took place in April at SF3 (46 ind.m^-3^) and the minimum was observed in November at SF3 (2 ind.m^-3^). Lastly, for Tunicata, the greatest abundance was observed in April at SF1 (406 ind.m^-3^), while the smallest was in November at SF2 (16 ind.m^-3^) (Fig. 5).

### Seasonal abundance of gelatinous species

At Habibas Islands, amongst the cnidarians, *Pelagianoctiluca* had the highest abundance with 24.53 (± 57.82) ind.m^-3^ in spring and 3.85 (± 4.4) ind.m^-3^ in summer (Table 2). Next, *Muggiaeaatlantica* displayed an abundance of 27.27 (± 21.02) ind.m^-3^ in spring and 4.12 (± 3.15) ind.m^-3^ in summer. Moreover, *Abylopsistetragona* showed an abundance of 5.4 (± 6.37) ind.m^-3^ in spring and 7.98 (± 2.01) ind.m^-3^ in summer. Additionally, *Lensiasubtiloides* had an abundance of 6.27 (± 9.78) ind.m^-3^ in spring, while *Lensiasubtilis* displayed an abundance of 2.12 (± 2.38) ind.m-^3^ in spring. *Aglaurahemistoma* had an abundance of 0.55 (± 0.25) ind.m^-3^ in summer, whereas *Clytiahemisphaerica* had an abundance of 0.19 (± 0.31) ind.m^-3^ in spring. Similarly, *Obeliaspp* displayed an abundance of 0.4 (± 0.32) ind.m^-3^ in spring. Finally, *Mitrocomiumcirratum*, *Rhopalonemavelatum* and *Solmundellabitentaculata* had lower abundances, with 0.13 (± 0.33) ind.m^-3^ in spring and 0.13 (± 0.33) ind.m^-3^ in summer, 0.13 (± 0.33) ind.m^-3^ in spring and 0.45 (± 0.31) ind.m^-3^ in summer and 0.48 (± 0.26) ind.m^-3^ in spring and 0.29 (± 0.28) ind.m^-3^ in summer, respectively.

Amongst the chaetognaths, *Flaccisagittaenflata* had a higher abundance of 15.37 (± 12.47) ind.m^-3^ in spring and 24.75 (± 18.11) ind.m^-3^ in summer. In contrast, *Parasagittafriderici* displayed an abundance of 0.95 (± 1.11) ind.m^-3^ in spring and 2.65 (± 1.98) ind.m^-3^ in summer.

Amongst the tunicates, *Thaliademocratica* displayed a high abundance of 88.92 (± 109.21) ind.m^-3^ in May. In addition, *Oikopleuralongicauda* had an abundance of 75.35 (± 42.86) ind.m^-3^ in May and 15.22 (± 12.75) ind.m^-3^ in July. Similarly, *Doliolumnationalis* showed a high abundance with 21.88 (± 7.27) ind.m^-3^ in May and 23.27 (± 16.88) ind.m^-3^ in July. *Oikopleurafusiformis* exhibited an abundance of 43.57 (± 30.1) ind.m^-3^ in May and 1.55 (± 1.95) ind.m^-3^ in July. *Oikopleuradioica* displayed an abundance of 18.42 (± 14.61) ind.m^-3^ in May and 1.6 (± 1.26) ind.m^-3^ in July. Moreover, *Fritillariapellucida* had an abundance of 32.73 (± 13.19) ind.m^-3^ in May and 3.38 (± 4.85) ind.m^-3^ in July. Lastly, *Fritillariaformica* showed an abundance of 3.85 (± 7.13) ind.m^-3^ in May and 1.17 (± 1.04) ind.m^-3^ in July.

Amongst the Mollusca, *Heliconoidesinflatus* exhibited the highest abundance with 2.38 (± 1.23) ind.m^-3^ in spring and 0.53 (± 0.43) ind.m^-3^ in summer. Following, *Limacinatrochiformis* showed an abundance of 3.17 (± 2.61) ind.m^-3^ in spring and 6.7 (± 6.33) ind.m^-3^ in summer. In contrast, *Cavoliniainflexa* and *Creseisvirgula* presented lower abundances of 0.1 (± 0.24) ind.m^-3^ and 0.3 (± 0.5) ind.m^-3^ in spring, respectively and were not observed in summer. Additionally, *Cliopolita* showed an abundance of 0.8 (± 0.98) ind.m^-3^ in spring and was not observed in summer. Overall, the Mollusca showed lower abundances compared to other groups, with *Heliconoidesinflatus* and *Limacinatrochiformis* being the most abundant species.

At Sidi Fredj, significant seasonal variations were observed in cnidarian species abundances (Table 2). The most abundant species were *Abylopsistetragona* 7.87 (± 4.54) ind.m^-3^, *Lensiasubtilis* 0.9 (±0.6) ind.m^-3^, *Muggiaeaatlantica* 0.9 (± 0.52) ind.m^-3^, *Rhopalonemavelatum* 0.29 (± 0.12) ind.m^-3^, *Aglaurahemistoma* 0.27 (± 0.46) ind.m^-3^ and *Solmundellabitentaculata* 0.27 (± 0.46) ind.m^-3^. Some species showed significant increases in abundance over the months, such as *Muggiaeaatlantica* which increased considerably from 0.9 (± 0.52) ind.m^-3^ in November to 44.17 (± 26.05) ind.m^-3^ in March, then to 63.53 (± 14.56) ind.m^-3^ in April, before decreasing slightly to 22.7 (± 11.06) ind.m^-3^ in July. *Lensiasubtilis* also showed a significant increase in abundance between November and April, from 0.9 (± 0.6) ind.m^-3^ to 24.07 (± 7.53) ind.m^-3^. On the other hand, some species showed significant decreases in abundance over the months, such as *Aglaurahemistoma* which decreased from 0.27 (± 0.46) ind.m^-3^ in November to 0.21 (± 0.05) ind.m^-3^ in July. The other cnidarian species did not show significant changes in abundance or showed less pronounced variations over the months.

The Chaetognatha group showed varying abundance levels amongst the different species studied at Sidi Fredj. *Flaccisagittaenflata* emerged as the most abundant species, with its abundance increasing from 25.8 (± 13.25) ind.m^-3^ in November to 122.67 (± 35.5) ind.m^-3^ in July. *Parasagittafriderici* was the second most abundant species, with numbers ranging between 1.4 (± 1.42) ind.m^-3^ in November and 23.07 (± 24.09) ind.m^-3^ in July. In comparison, the other species, such as *Mesosagittaminima*, *Pterosagittadraco* and *Pseudosagittalyra*, exhibited much lower abundances.

The Tunicata group exhibited a range of abundance levels for the various species studied. *Doliolumnationalis* was the most abundant species, with its abundance increasing from 4.63 (± 1.96) ind.m^-3^ in November to 66.07 (± 67.49) ind.m^-3^ in July. *Fritillariapellucida* was another abundant species, with numbers ranging from 23.63 (± 26.88) ind.m^-3^ in March to 98.6 (± 27.37) ind.m^-3^ in April. *Oikopleuralongicauda* also showed a significant presence, with its abundance increasing from 6.3 (± 0.69) ind.m^-3^ in November to 35.5 (± 50.29) ind.m^-3^ in July. Other species, such as *Fritillariaformica*, *Fritillariafraudax* and *Oikopleurarufescens*, showed relatively lower abundances in comparison to the aforementioned species.

In the Mollusca group, a range of abundance levels was observed amongst the different species. *Limacinatrochiformis* stood out as the most abundant species, with its abundance increasing from 1.3 (± 1.35) ind.m^-3^ in November to 26.23 (± 8.95) ind.m^-3^ in April and then decreasing to 8.3 (± 6.58) ind.m^-3^ in July. *Heliconoidesinflatus* ranked as the second most abundant species, with its abundance ranging from 0.5 (± 0.46) ind.m^-3^ in November to 10.7 (± 12.3) ind.m^-3^ in July. The other species, such as *Cavoliniainflexa*, *Cliopolita* and *Creseisvirgula*, had much lower abundances in comparison (Table 2).

**Figure 6.**
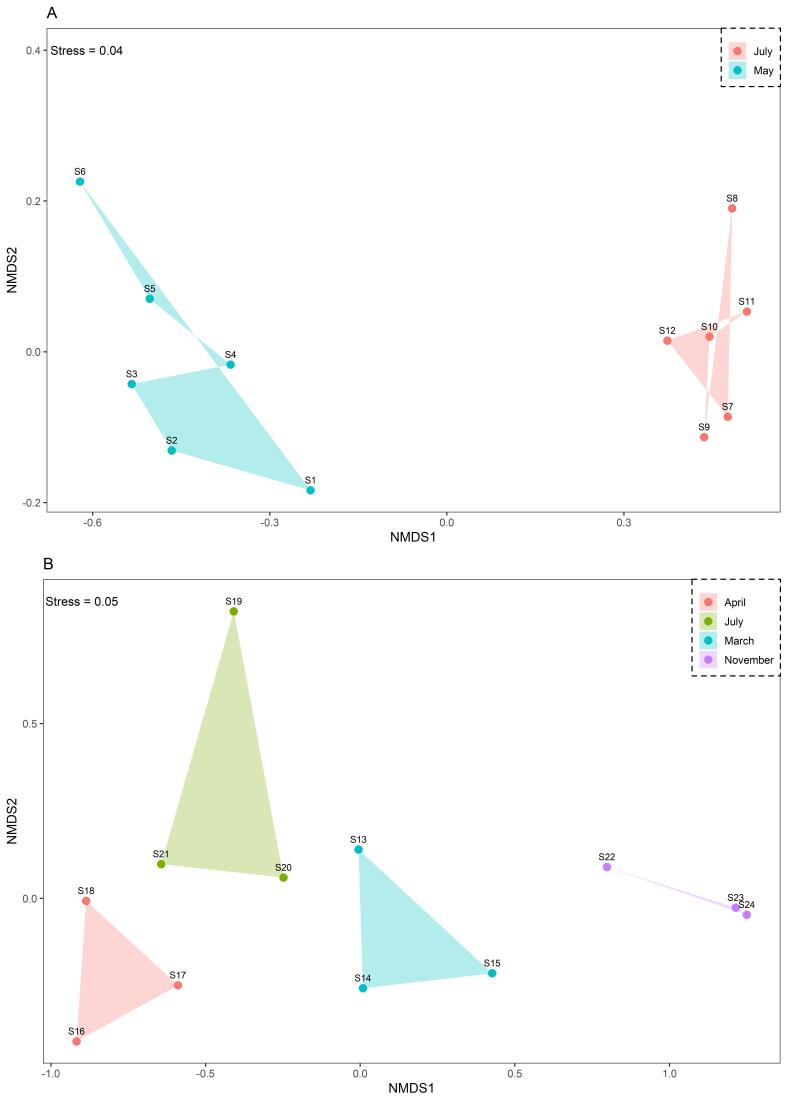
non-Metric Multidimensional Scaling ordination (nMDS), based on sample abundances data showing the distribution of gelatinous zooplankton samples in the Algerian coast. **A**: Distribution of zooplankton in Habibas Islands; **B**: Distribution of zooplankton in Sidi Fredj

The nMDS analysis emphasises the distinct groupings and unveils the dissimilarity between the Habibas Islands and Sidi Fredj (Fig. 6). Table 3 further supports these observations by comparing ANOSIM test results across various time periods and regions, with a particular focus on the Habibas Islands and Sidi Fredj. The evaluation of these findings relies on the R statistic value and the corresponding significance level (P-value). A significant difference in the ecological community structure is observed within the Habibas Islands between May and July, as evidenced by an R statistic value of 1 and a significance level of 0.002. This indicates a notable dissimilarity between the two months. In contrast, within Sidi Fredj, no significant differences are detected for the provided month combinations. All combinations display a significance level of 0.1, suggesting that the observed differences between these months are likely not significant. When comparing the ecological community structures of the Habibas Islands and Sidi Fredj, several month combinations reveal moderately significant differences. For instance, the month pairings of May - March, May - April, May - July and May - November exhibit R statistic values close to or equal to 1, denoting a significant difference between these months across both regions. Other month combinations, such as July - March and July - April, also present R statistic values near 1, implying a significant difference between these months for the two regions. Lastly, the July - July combination yields an R statistic value of 0.938, signifying a significant difference, albeit less pronounced compared to the other combinations.

### Relationship between species and environmental factors

**Figure 7.**
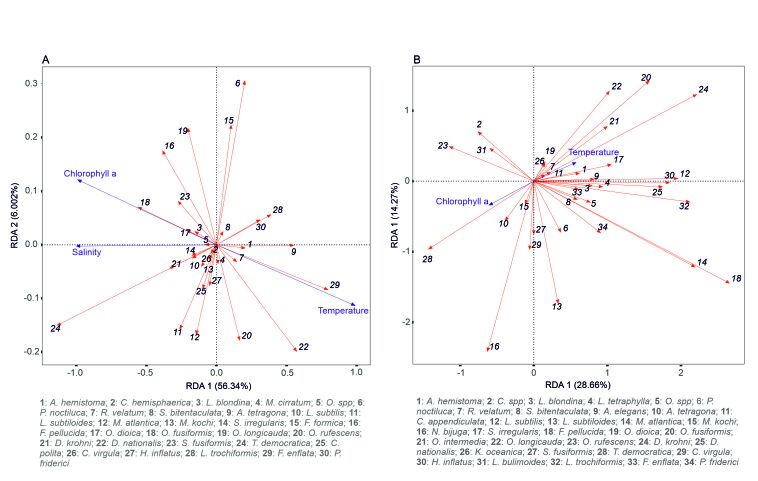
Redundancy analysis ordination plot of gelatinous species (red arrows) with environmental variables (blue arrows) for zooplankton samples. **A**: Habibas Islands; **B**: Sidi Fredj

In the Habibas Islands (Fig. 7 A), the redundancy analysis plot reveals relationships between different marine species and environmental variables. Cnidarian species, like *P.noctiluca*, *S.bitentaculata*, *A.hemistoma*, *R.velatum* and *A.tetragona*, show positive correlations with temperature, while *P.noctiluca* is negatively correlated with chlorophyll a and salinity. For Tunicata, Oikopleuridae species and *D.nationalis* are positively related to temperature, whereas *F.pellucida* is negatively related. Thaliacea species are positively correlated with chlorophyll a and salinity, while *D.nationalis* is negatively related to salinity. Lastly, the pelagic mollusc *L.trochiformis* is positively correlated with temperature, while *C.virgula* is negatively correlated with temperature, but positively related to salinity and chlorophyll a.

In Sidi Fredj (Fig. 7 B), the redundancy analysis plot provides insight into the relationships between various marine species and environmental variables. Tunicata species, including *O.logicauda*, *O.fusiformis*, *O.intermedia*, *F.pellucida*, *D.krohni* and *D.nationalis*, show a positive correlation with temperature, indicating that these species may thrive in warmer conditions. In contrast, *T.democratica* and *O.rufescens*, both belonging to the Tunicata group, demonstrate a correlation with chlorophyll a, suggesting that these species might be influenced by the availability of nutrients or phytoplankton in the water. In addition to Tunicata, several cnidarian species, such as *M.atlantica*, *P.noctiluca*, *S.bitentaculata*, *L.tetraphylla*, *A.elegans*, *L.subtilis*, *S.irregularis*, *A.hemistoma* and *L.blondina*, also exhibit positive correlations with temperature. This suggests that these species may also prefer or be more abundant in warmer conditions. Moreover, the RDA plot reveals that chaetognaths, a group of predatory marine worms and molluscs, including *H.inflatus* and *L.trochiformis*, also display positive correlations with temperature.

## Discussion

The purpose of this research was to broaden our understanding of gelatinous zooplankton along the central and western Algerian coast. This was achieved by examining the temporal patterns, taxonomy, occurrence and community structure of these organisms in the Habibas Islands and Sidi Fredj region, taking into account environmental factors. In total, 48 gelatinous zooplankton species were identified, most of which have been previously reported along the Algerian coast and in the western Mediterranean. This diverse group of species represents a cosmopolitan fauna, with some species being characteristic of Atlantic waters, such as *M.atlantica* and *L.subtiloides* (*Gamulin and Kršinic 1993*). Other species, like *P.friderici*, *P.draco*, *C.affinis* and *K.oceanica*, also inhabit the Atlantic (Ghirardelli and Gamulin 2014). Comparing our findings to past research in the south-western Mediterranean's Alboran Sea, 59 species (Dallot et al. 1988) and 58 species (Mills et al. 1996) have been reported. In contrast, lower gelatinous zooplankton biodiversity was found in Algiers Bay (31 species; Seguin (1973)) and Annaba Bay (19 species; Ounissi et al. (2016)) along the Algerian coast. When evaluating our results against other Mediterranean regions, we discovered that the gelatinous zooplankton biodiversity in Bizerte Bay, Tunisia, comprised 48 species (Touzri et al. 2012). In the Adriatic Sea, over 57 species were identified (Batistić et al. 2004), which decreased to 44 species (Pestorić et al. 2016). In contrast, the species richness of gelatinous zooplankton on the Egyptian (Zakaria 2006) and Lebanese coasts (Lakkis 2013) was higher, with 67 and 151 species, respectively. It is crucial to acknowledge that the differences in gelatinous zooplankton biodiversity between our study and previous ones can be ascribed to various factors, such as the study sites along the Mediterranean coast, the collection period and the sampling effort, which can all influence the distribution of zooplankton.

Total abundances of zoological groups at Habibas Islands and Sidi Fredj vary from one month to another, not exceeding 500 ind.m^-3^, due to specific environmental factors in the region. The sampling stations are located in the Algerian Basin, where the Atlantic surface waters are offset by a westward countercurrent of deep Mediterranean waters (Huertas et al. 2012), creating oligotrophic conditions that limit the productivity of zooplankton communities (Hafferssas and Seridji 2010, Khames and Hafferssas 2019, Sola et al. 2019). These conditions lead to a decrease in nutrient concentration from west to east in the Mediterranean. Additionally, high summer temperatures and oligotrophic conditions hinder the development of zooplankton in the southwest Mediterranean (Kherchouche and Hafferssas 2020). During the spring season, the bloom of phytoplankton was often linked to the highly enriched environment in nutrients, caused by vertical mixing of the water column. Favourable conditions, including warmer temperatures, increased light levels and the presence of nutrients, are responsible for the spring phytoplankton bloom (Batistić et al. 2019). Such conditions often lead to the bloom of small, fast-growing gelatinous taxa (i.e. siphonophores and tunicates), followed by larger ones (Sola et al. 2019). This variation in gelatinous zooplankton populations observed in the study can be attributed to the presence of specific species, such as *Pelagianoctiluca*, *Muggiaeaatlantica*, *Abylopsistetragona*, *Lensiasubtilis*, and *Aglaurahemistoma*, as well as thaliaceans like *Thaliademocratica*, *Fritillariapellucida*, *Oikopleuralongicauda* and *Doliolumnationalis*. Chaetognaths, including *Flaccisagittaenflata* and *Parasagittafriderici*, also contributed to this variation. These species emerged as the most abundant gelatinous taxa, consistent with prior research conducted in the Algerian Basin (Khames and Hafferssas 2019, Kherchouche and Hafferssas 2020). This observation not only highlights the significance of these particular species within the local ecosystem, but also connects the findings with similar patterns reported in other Mediterranean regions, such as the Ligurian Sea (Licandro et al. 2006, Licandro et al. 2012), the Catalan Sea (Guerrero 2017), the Adriatic Sea (Batistić et al. 2004, Pestorić et al. 2016) and the Tunisian coast (Touzri et al. 2010, Touzri et al. 2012). Collectively, these results emphasise the consistent pattern of diverse and abundant gelatinous zooplankton species across various Mediterranean regions.

In this study, redundancy analysis highlights a strong correlation between gelatinous zooplankton and temperature. As a result, temperature emerges as the most influential explanatory variable for certain cnidarian species. High water temperatures positively impact various stages of cnidarian reproduction, which can lead to rapid population growth and persistence throughout the winter season (Boero et al. 2008). For example, siphonophores prefer the warm and temperate waters of the neritic region (Mapstone 2014). Our research reveals a significant association between *M.atlantica* and temperature, a pattern previously observed in other global ocean regions (Lo et al. 2014, Mapstone 2014). Population concentrations of *M.atlantica* are also related to salinity variability. Salinity is one of the factors regulating the distribution of *M.atlantica* in the global ocean (Licandro et al. 2012, Blackett et al. 2015, Guerrero et al. 2016). Salinity appears to be a determining factor in the reproductive cycle of the Muggiaea genus (Carré and Carré 1991). Thermal variability is also associated with the distribution of *A.hemistoma* along the Algerian coasts. Our results support those obtained by other researchers (Guerrero et al. 2016, Flores Coto et al. 2016) and extend to other species such as *P.noctiluca*, *R.velatum*, *L.tetraphylla*, *Obeliaspp*, *L.blondina* and *A.tetragona*. Comparable trends have been documented by Buecher 1999, Sanvicente-Añorve et al. 2009, Pavez et al. 2010 and Licandro et al. 2012. Moreover, temperature fluctuations influence the developmental cycle of Medusozoa (Goy et al. 1989). As an example, the larval growth of the species *P.noctulica* is directly proportional to rising temperatures (Morand et al. 1992, Richardson et al. 2009).

The warming of the epipelagic layer waters has been identified as having an impact on the behaviour of thaliaceans. *D.nationalis* is one of the species that exemplifies this trend, as noted by Braconnot and Dallot (1995), Deibel (1998) and Deibel and Paffenhöfer (2009). Gibson and Paffenhöfer (2002) observed two peaks in *D.nationalis* abundance in spring and summer, corresponding to an increase in nutrient resources. It is essential to emphasise that the filtration rate of *T.democratica* is closely linked to temperature variations (Stone and Steinberg 2016). Furthermore, the concentration of chlorophyll a significantly affects the growth, abundance and spatial distribution of *T.democratica* (Alldredge and Madin 1982). Regarding appendicularians, Fenaux et al. (1998) noted that warmer waters favour the growth of populations, such as *O.longicauda* and *O.fusiformis*, while Siokou-Frangou et al. (1998) found that *F.pellucida* thrives in winter and spring. These observations are consistent with other Mediterranean studies, such as those by Fenaux (1967) and Fernández de Puelles et al. (2003). Finally, López-Urrutia et al. (2003) and Licandro et al. (2006) emphasised that phytoplankton blooms increase the production levels of certain plankton species, which can contribute to their proliferation in the northwest Mediterranean.

Numerous studies (Dallot et al. 1988, Pierrot-Bults and Nair 1991, Fernández de Puelles et al. 2003, Daponte et al. 2004, Batistić et al. 2007) indicate that seasonal variability significantly impacts Chaetognath populations, such as *F.enflata* and *S.friderici*. Along the Algerian coast, warmer waters promote the growth of *F.enflata* populations. This widespread species is predominantly found in warm temperate waters (Pierrot-Bults and Nair 1991). During the summer season, it dominates in the north-western Mediterranean (Andreu 1990), in the Alboran Sea (Dallot et al. 1988) and along the Lebanese coasts (Lakkis 2013). This observation is not only applicable to Chaetognaths, but also holds true for planktonic molluscs. Specifically, the spring and summer seasons foster the growth of *L.trochiformis* and *H.inflatus* populations. Consequently, these species are found in substantial numbers in the Adriatic Sea during the period from February to September (Pestorić et al. 2016). Moreover, they exhibit a high sensitivity to temperature fluctuations, as highlighted by Rampal (1975).

## Conclusion

The recently conducted study sheds light on the gelatinous zooplankton communities present along the Algerian coast. The research indicates that the Habibas Islands and Sidi Fredj exhibit a diverse range of species, with seasonal variations in both species richness and abundance. The study also emphasises the role of abiotic environmental conditions, including temperature, salinity and chlorophyll a levels, in regulating the population dynamics of these communities. The results underscore the significance of these factors in shaping the distribution and abundance of gelatinous zooplankton, which are essential components of marine ecosystems. This study contributes to a better understanding of the dynamics of gelatinous zooplankton communities in the south-western Mediterranean Sea, emphasising the importance of continued monitoring of these communities.

The implications of this study are critical for various stakeholders, including government officials, managers and fisheries scientists. The findings provide valuable information for designing policies and management strategies for sustainable fisheries, taking into account seasonal variations in zooplankton abundance and diversity. Government officials can use the results to create policies that aim to conserve the seasonal abundance of zooplankton and maintain healthy fish populations. Managers of fisheries can use this information to develop sustainable fishing practices that consider the seasonal variations in zooplankton abundance. Protective measures could be put in place during periods when zooplankton abundance is low to ensure adequate reproduction and growth of fish populations. These policies could include fishing quotas, marine protected areas or seasonal fishing restrictions.

Finally, our study may also benefit the scientific community studying fish. By understanding seasonal patterns in zooplankton abundance, researchers can better understand trophic interactions between fish and their prey, as well as the impacts of climate and environmental changes on marine ecosystems.

## Figures and Tables

**Table 1.** Sampling of gelatinous zooplankton ion the Algerian coast.

**Areas**	**Stations**	**Longitudes**	**Latitudes**	**Season**	**Sampling dates**
**Habibas Islands**	HI1	1°10'W	35°37'42''	Spring	13/05/2012
				Summer	12/07/2012
	HI2		35°40'00"	Spring	13/05/2012
				Summer	12/07/2012
	HI3		35°41'00''	Spring	13/05/2012
				Summer	12/07/2012
	HI4	1°8'W	35°44'30"	Spring	13/05/2012
				Summer	12/07/2012
	HI5		35°44'48''	Spring	13/05/2012
				Summer	12/07/2012
	HI6		35°45'24''	Spring	13/05/2012
				Summer	12/07/2012
**Sidi Fredj**	SF1	2°50'E	36°47'24''	Autumn	18/11/2012
				Winter	04/03/2013
				Spring	16/04/2013
				Summer	11/07/2013
	SF2		36°48'12''	Autumn	18/11/2012
				Winter	04/03/2013
				Spring	16/04/2013
				Summer	11/07/2013
	SF3		36°49'10''	Autumn	18/11/2012
				Winter	04/03/2013
				Spring	16/04/2013
				Summer	11/07/2013

**Table 2.** Gelatinous zooplankton mean abundance (Abd) ind.m^−3^ and standard deviation (± SD) in Habibas Islands (HI) and Sidi Fredj (SF)

**Taxa**	**Habibas Islands**		**Sidi Fredj**			
	**May**	**July**	**November**	**March**	**April**	**July**
Cnidaria						
*Abylopsistetragona* (Otto, 1823)	5.4 ± 6. 37	7.98 ± 2.01	7.87 ± 4.54	15.1 ± 7.47	7.4 ± 2.6	3.97 ± 3.25
Agalmaelegans (Sars, 1846)					1 ± 1.73	0.4 ± 0.69
*Aglaurahemistoma* Péron & Lesueur, 1810		0.55 ± 0.25	0.27 ± 0.46	0.16 ± 0.14	1.23 ± 1.36	0.21 ± 0.05
*Chelophyesappendiculata* (Eschscholtz, 1829)				0.2 ± 0.35		1 ± 1.25
*Clytiahemisphaerica* (Linnaeus, 1767)	0.19 ± 0.31				0.27 ± 0.46	
*Clytiaspp* Lamouroux, 1812			0.8 ± 1.06		0.27 ± 0.46	
*Eucheilotaparadoxica* Mayer, 1900						0.27 ± 0.46
*Hydractiniasp* Van Beneden, 1844						0.27 ± 0.46
*Lensiasubtilis* (Chun, 1886)	2.12 ± 2.38		0.9 ± 0.6	5 ± 1.82	18.17 ± 7.53	24.07 ± 14.99
*Lensiasubtiloides* (Lens & van Riemsdijk, 1908)	6.27 ± 9.78			2.27 ± 1.59	0.3 ± 0.3	0.8 ± 1.39
*Liriopetetraphylla* (Chamisso & Eysenhardt, 1821)				0.32 ± 0.42	0.64 ± 0.28	0.27 ± 0.46
*Lizziablondina* Forbes, 1848	2.16 ± 3.7				0.53 ± 0.46	
*Mitrocomiumcirratum* Haeckel, 1879	0.13 ± 0.33	0.13 ± 0.33				
*Muggiaeaatlantica* Cunningham, 1892	27.27 ± 21.02	4.12 ± 3.15	0.9 ± 0.52	44.17 ± 26.05	63.53 ± 14.56	22.7 ± 11.06
*Muggiaeakochii* (Will, 1844)	1.18 ± 1.78		0.2 ± 0.35	0.2 ± 0.35	0.4 ± 0.69	
*Nanomiabijuga* (Delle Chiaje, 1844)				4.9 ± 0.69		
*Obeliaspp* Péron & Lesueur, 1810	0.4 ± 0.32			0.27 ± 0.46	0.61 ± 0.32	
*Pelagianoctiluca* (Forsskål, 1775)	24.53 ± 57.82	3.85 ± 4.4		1.95 ± 2.09	10.93 ± 18.94	
*Phialellaquadrata* (Forbes, 1848)						0.27 ± 0.46
*Proboscidactylaornata* (McCrady, 1859)					0.27 ± 0.46	
*Rhopalonemavelatum* Gegenbaur, 1857	0.13 ± 0.33	0.45 ± 0.31	0.29 ± 0.12	0.93 ± 1.62	0.4 ± 0.21	0.45 ± 0.32
*Solmarissp* Haeckel, 1879						
*Solmundellabitentaculata* (Quoy & Gaimard, 1833)	0.48 ± 0.26	0.29 ± 0.28	0.27 ± 0.46	0.37 ± 0.4	1.23 ± 0.88	0.27 ± 0.46
*Sphaeronectesirregularis* (Claus, 1873)	1.7 ± 1.49		0.3 ± 0.52	1 ± 0.92	0.9 ± 0.79	2.8 ± 0.92
Chaetognatha						
*Flaccisagittaenflata* (Grassi, 1881)	15.37 ± 12.47	24.75 ± 18.11	25.8 ± 13.25	61.07 ± 30.64	76.17 ± 49.93	122.67 ± 35.5
*Mesosagittaminima* (Grassi, 1881)				0.03 ± 0.06		
*Parasagittafriderici* (Ritter-Záhony, 1911)	0.95 ± 1.11	2.65 ± 1.98	1.4 ± 1.42	11.43 ± 1.67	8.27 ± 3.71	23.07 ± 24.09
*Pseudosagittalyra* (Krohn, 1853)				0.07 ± 0.12		
*Pterosagittadraco* (Krohn, 1853)				0.1 ± 0.17		
Tunicata						
*Cyclosalpaaffinis* (Chamisso, 1819)	0.05 ± 0.12					
*Doliolinakrohni* Herdman, 1888	7.05 ± 5.36		0.6 ± 0.52	0.6 ± 0.6	39.73 ± 47.61	8.5 ± 11.62
*Doliolumnationalis* Borgert, 1893	21.88 ± 7.27	23.27 ± 16.88	4.63 ± 1.96	12.73 ± 2.05	175.47 ± 23.67	66.07 ± 67.49
*Fritillariaformicatuberculata* Lohmann in Lohmann & Buckmann, 1926	3.85 ± 7.13	1.17 ± 1.04	0.1 ± 0.17	0.2 ± 0.35		
*Fritillariafraudax* Lohmann, 1896			0.1 ± 0.17			
*Fritillariapellucida* (Busch, 1851)	32.73 ± 13.19	3.38 ± 4.85	0.2 ± 0.35	23.63 ± 26.88	98.6 ± 27.37	2.6 ± 1.25
*Kowalevskiaoceanica* Lohmann, 1899						0.6 ± 1.04
*Oikopleuradioica* Fol, 1872	18.42 ± 14.61	1.6 ± 1.26	2.3 ± 0.62	2.97 ± 4.63	5.03 ± 2.54	1.2 ± 1.04
*Oikopleurafusiformis* Fol, 1872	43.57 ± 30.1	1.55 ± 1.95	1.4 ± 1.65	0.1 ± 0.17	4.83 ± 2.8	12.03 ± 15.28
*Oikopleuraintermedia* Lohmann, 1896					0.4 ± 0.46	2.77 ± 2.46
*Oikopleuralongicauda* (Vogt, 1854)	75.35 ± 42.86	15.22 ± 12.75	6.3 ± 0.69	2.37 ± 3.06	30.4 ± 14.57	35.5 ± 50.29
*Oikopleurarufescens* Fol, 1872	0.95 ± 1.33	1.8 ± 1.83	2.6 ± 0.46	0.8 ± 0.92	0.4 ± 0.46	2.17 ± 3.25
*Salpafusiformis* Cuvier, 1804	5.78 ± 10.43			0.4 ± 0.35	0.1 ± 0.17	
*Thaliademocratica* (Forskål, 1775)	88.92 ± 109.21		2.27 ± 2.44	3.57 ± 3.55	0.4 ± 0.69	
Mollusca						
*Cavoliniainflexa* (Lesueur, 1813)	0.1 ± 0.24					
*Cliopolita* Pelseneer, 1888	0.8 ± 0.98					
*Creseisvirgula* (Rang, 1828)	0.3 ± 0.5			0.8 ± 0.92	0.1 ± 0.17	
*Heliconoidesinflatus* (d'Orbigny, 1835)	2.38 ± 1.23	0.53 ± 0.43	0.5 ± 0.46	2.27 ± 2.15	6.5 ± 4.62	10.7 ± 12.3
*Limacinabulimoides* (d'Orbigny, 1835)			0.6 ± 0.79			
*Limacinatrochiformis* (d'Orbigny, 1835)	3.17 ± 2.61	6.7 ± 6.33	1.3 ± 1.35	4.07 ± 2.39	26.23 ± 8.95	8.3 ± 6.58

**Table 3.** ANOSIM pairwise comparison of gelatinous zooplankton abundance in the Algerian coast significance levels; *: <0.05 **: < 0.01; ***: < 0.001)

**Regions**	**Months**	**Seasons**	**R statistic**	**Significance level** %
Habibas islands - Habibas islands	May - July	Spring- Summer	1	0.002 ***
Sidi Fredj - Sidi Fredj	Mars - April	Winter - Spring	1	0.1 ns
Sidi Fredj - Sidi Fredj	Mars - July	Winter - Summer	0.704	0.1 ns
Sidi Fredj - Sidi Fredj	Mars - November	Winter - Autumn	1	0.1 ns
Sidi Fredj - Sidi Fredj	April - July	Spring - Summer	0.519	0.1 ns
Sidi Fredj - Sidi Fredj	April - November	Spring - Autumn	1	0.1 ns
Sidi Fredj - Sidi Fredj	July - November	Summer - Autumn	0.778	0.1 ns
Habibas islands - Sidi Fredj	May - March	Spring - Winter	0.981	0.012 **
Habibas islands - Sidi Fredj	May - April	Spring - Spring	0.994	0.012 **
Habibas islands - Sidi Fredj	May - July	Spring - Summer	1	0.012 **
Habibas islands - Sidi Fredj	May - November	Spring - Autumn	0.981	0.012 **
Habibas islands - Sidi Fredj	July - March	Summer - Winter	1	0.012 **
Habibas islands - Sidi Fredj	July - April	Summer - Winter	0.988	0.012 **
Habibas islands - Sidi Fredj	July - July	Summer - Summer	0.938	0.012 **
Habibas islands - Sidi Fredj	July - November	Summer - Autumn	0.981	0.012 **
